# A person-centered approach to characterizing longitudinal ambulatory impairment in Parkinson's disease

**DOI:** 10.1038/s41598-024-62179-9

**Published:** 2024-05-20

**Authors:** Farren B. S. Briggs, Douglas D. Gunzler, Steven A. Gunzler

**Affiliations:** 1 Department of Public Health Sciences, University of Miami Miller School of Medicine, 1140 NW 14th St, 912 Don Soffer Clinical Research Center, Miami, FL 33136 USA; 2 Center for Health Care Research and Policy, Case Western Reserve University School of Medicine, Cleveland, OH USA; 3 Department of Population and Quantitative Health Sciences, Case Western Reserve University School of Medicine, Cleveland, OH USA; 4 Neurological Institute, University Hospitals Cleveland Medical Center and Case Western Reserve University School of Medicine, Cleveland, OH USA; 5 Department of Neurology, Case Western Reserve University School of Medicine, Cleveland, OH USA

**Keywords:** Ambulatory impairment, Parkinson’s disease, Latent class growth analysis, Patient reported outcome, Trajectories, Parkinson's disease, Epidemiology

## Abstract

Loss of ambulation is common and highly variable in Parkinson’s disease (PD), and poorly understood from the perspectives of those with PD. Gaining insights to the anticipated perceived trajectories and their drivers, will facilitate patient-centered care. Latent class growth analysis, a person-centered mixture modelling approach, was applied to 16,863 people with PD stratified by early (N = 8612; < 3 years), mid (N = 6181; 3–10 years) and later (N = 2070; > 10 years) disease to discern clusters with similar longitudinal patterns of self-reported walking difficulty, measured by EuroQoL 5D-5L that is validated for use in PD. There were four clusters in early and mid-disease strata, with a fifth identified in later disease. Trajectories ranged from none to moderate walking difficulty, with small clusters with severe problems. The percentage of subjects with moderate (early = 17.5%, mid = 26.4%, later = 32.5%) and severe (early = 3.8%, mid = 7.4%, later = 15.4%) walking difficulty at baseline increased across disease duration groups. The trajectories tended to be stable with variability in moderate and severe groups. Across strata, clusters with moderate to severe problems were associated with more severe impairment, depression, anxiety, arthritis, higher BMI, lower income, and lower education, but no consistent race or gender differences. The findings reveal distinct longitudinal patterns in perceived difficulties in walking in PD.

## Introduction

Difficulty in walking (also referred to as ambulatory impairment) is a common and visible impairment experienced by people with Parkinson's disease (PWP), and it is driven by a diverse collection of symptoms (e.g. start hesitation, shuffling gait, freezing, festination, propulsion, and difficulty in turning)^1^. It is also a prominent driver of lower quality of life (QoL) in Parkinson’s disease (PD) and it is associated with poor health outcomes, increased depressive symptoms, more frequent falls, loss of independence, decreased social participation, and greater interruptions of daily activities^1–5^. Unfortunately, there are substantial fluctuations in the severity of the underlying symptoms and in the accrual of neurological deficits that impair walking in PWP; for example, changes in gait is highly variable and appears unpredictable in PWP over time^6^. This poses a significant challenge for successful patient-centered care, including tailoring clinical and rehabilitation care, prognostication, and developing long-term self-management strategies, as well as a challenge for defining robust endpoints in clinical and observational research.

Patient-reported outcome (PRO) measures capture the lived experiences of patients, including meaningful and nuanced changes in health-related QoL, and over time they inherently reflect patients’ shifting priorities for daily living. There are several PD-specific PROs for mobility (i.e., MDS-UPDRS Part II); however, these instruments do not readily map to generic PROs which impedes comparisons with the general population and subpopulations where ambulatory impairment is also seemingly unpredictable (i.e., persons with multiple sclerosis). Also, it has been noted that the perceptions (and/or key health priorities) of PWP may evolve with their disease course^7,8^; e.g., in a qualitative study of functional mobility, the perceptions of people in the early-stages of PD were more aligned with neurologists while those in more advanced-stages were closer to physiotherapists^9^. Another important factor is the underlying heterogeneity in ambulatory impairment in PWP. Prior studies have only described relationships for the *average* change in measures of gait and walking speed. No study has yet described the likely intrinsic subgroups of PWP who exhibit similar longitudinal ambulatory trajectories over time, based in part on the combination and severity of underlying symptoms that evolve as PD progresses. Fortunately, latent class growth analysis (LCGA) is a data-driven approach that can identify these naturally occurring subgroups with distinct growth trajectories within a larger sample and it has been successfully used to discern distinct subgroups of PWP with similar longitudinal pain (measured by a generic PRO measure) trajectories^10–12^. Thus, several knowledge gaps may be addressed by leveraging LCGA to longitudinally model ambulatory impairment in PWP using a generic health-related QoL PRO, with considerations for disease duration.

The objective of the current retrospective cohort study is to describe longitudinal ambulatory impairment trajectories in PWP leveraging self-reported information as captured by the European Quality of Life (EuroQoL) Questionnaire 5 level version (EQ-5D-5L) is a generic health-related QoL instrument that has construct validity in diverse populations and in PWP^13–16^. We hypothesize EQ-5D-5L walking difficulties component will vary as a function of disease duration and that sociodemographic and clinical factors will be associated with assignment to distinct trajectories at each disease duration stage. We hope that by defining subgroups of PWP with shared perceived ambulatory impairment patterns, there is the potential to advance clinical/observational research and patient-centered care that can be readily compared to other populations.

## Methods

### Research ethics

This secondary data analysis of de-identified data was deemed as non-human subject research by the institutional review boards at Case Western Reserve University and The MetroHealth System, Cleveland, Ohio.

### Study design

Fox Insight (https://foxinsight.michaeljfox.org) is a virtual and ongoing longitudinal study of people aged 18 years or older, with and without PD, led by the Michael J. Fox Foundation^17^. It aims to facilitate discovery, validation, and reproducibility in PD PRO research, and includes several PROs, routine health and medical assessments, environmental exposure and healthcare preference questionnaires, with the option to provide biospecimens for genotyping^17^. PWP were recruited to participate in Fox Insight using a multi-prong strategy that included broad (public announcement of Fox Insight on CBS Sunday Morning), tailored (i.e. Facebook Campaign for “Late PD”, Google Search Engine Marketing Campaign for “Early PD”), and geotargeted digital marketing campaigns^18^. The longitudinal data used were obtained from Fox Insight Data Exploration Network (Fox DEN) on 10/14/2021 and leveraged to construct a retrospective cohort of PWP who had completed the EQ-5D-5L at least once (for up-to-date information visit https://foxinsight-info.michaeljfox.org/insight/explore/insight.jsp)^12^. PWP were defined as those self-reporting having a diagnosis of PD by a physician or health care professional (a video-based validation study observed strong agreement between self-reported diagnosis and clinician-determined diagnosis (kappa = 0.89, 95% CI 0.81, 0.97))^19^.

### Outcome

EQ-5D-5L measures perspectives on five domains, including self-care, usual activities, pain/discomfort, anxiety/depression, and walking difficulty^20^. The outcome of interest was the longitudinal data for the single-item component of the EQ-5D-5L that asks about experiencing any problems with walking (hereto referred to as ambulatory impairment and mobility PRO for brevity—we acknowledge that in general mobility encompasses a broader range of movements and activities that allow individuals to navigate their environment and here we are only focusing on self-reported difficulties in ambulation/walking). EQ-5D-5L was first deployed in 2017 and available under “Your Physical Experiences” in Fox DEN^17^. The mobility PRO is ordinal, measured on a 5-level Likert scale (0 = I have no problems in walking about, 1 = slight problems, 2 = moderate problems, 3 = severe problems, 4 = unable to walk about). There were 16,863 PD participants with EQ-5D-5L ambulatory impairment data at baseline and ≥ 1 additional follow-up survey, and who had an indicator value for number of years with PD (early: < 3 years [N = 8612 PWP], mid: 3–10 years [N = 6181 PWP], later: > 10 years [2070 PWP]). EQ-5D-5L may be completed at 6-month intervals; included subjects completed an average of 4.1 (SD 2.1) surveys. There were 11,838 (70%), 8557 (51%), 6029 (36%), 4257 (25%), 2736 (16%), 1475 (9%) and 554 (3%) PWP with ≥ 3, ≥ 4, ≥ 5, ≥ 6, ≥ 7, ≥ 8, and ≥ 9 entries, respectively. Note that the decrease in sample size over time is not necessarily a matter of loss-to-follow-up (left censoring), but also right censoring, reflecting the ongoing recruitment of PWP.

Only ≤ 9 observations per PWP were used for the stratified models for early and mid-disease duration, while ≤ 8 observations per PWP were used for the models for later disease duration to minimize data sparseness considering the total number of subjects endorsing each of the five mobility PRO categories at each follow-up time point. Consecutive responses for this PRO have high but incomplete concordance which mitigates concerns of redundancy and multicollinearity between in any two successive observations (Pearson correlation coefficient [PCC] = 0.59–0.82; similar patterns observed across disease duration strata early: PCC = 0.56–0.81, mid: PCC = 0.58–0.82, later: PCC = 0.53–0.80). It was important to stratify by disease duration as the accrual of ambulatory impairment in PWP is a function of disease function, and there are likely different rates at which impairment is accrued for a given length of disease, and lastly, perceptions of one’s disability may evolve with time^7–9^.

### Baseline variables

As we have previously described, the baseline sociodemographic variables incorporated included age, gender, race/ethnicity (non-white vs. white), education (1 = Less than high school degree, 2 = High school degree, 3 = Some college, 4 = Associate’s degree, 5 = Bachelor’s degree, 6 = Master’s degree, 7 = Doctoral degree), employment (retired, full-time, part-time, or unemployed; retired was the reference category for employment dummy variables in the multivariable regression models), income (1 =  < $20,000, 2 = $20,000–$34,999, 3 = $35,000–$49,999, 4 = $50,000–$74,999, 5 = $75,000–$99,999, 6 =  > $100,000), and body mass index (BMI)^12^. Self-reported clinical factors were included based on their hypothesized relationships with ambulatory impairment in PWP, and included binary indicators about current depression, anxiety, arthritis, and back pain duration and limitations (from “Your Current Health”); poor balance (from “Brief Motor Screen”), experiences of OFF episodes (from “Impact of OFF Episodes”), work in the past week (from “Work-related Activity”), trouble getting out of bed, a car seat, or a deep chair, walking and balance problems and freezing up (from “Your Movement Experiences”) and walking activities, light, moderate and strenuous sport and recreational activities and muscle strength (from “Your Physical Activities”)^17^. Military veteran status, actively taking prescription PD medication, and EQ-5D-5L pain component (ordinal items: 0 = no pain, 4 = extreme pain) were also included.

### Statistical analyses

**Figure 1:**
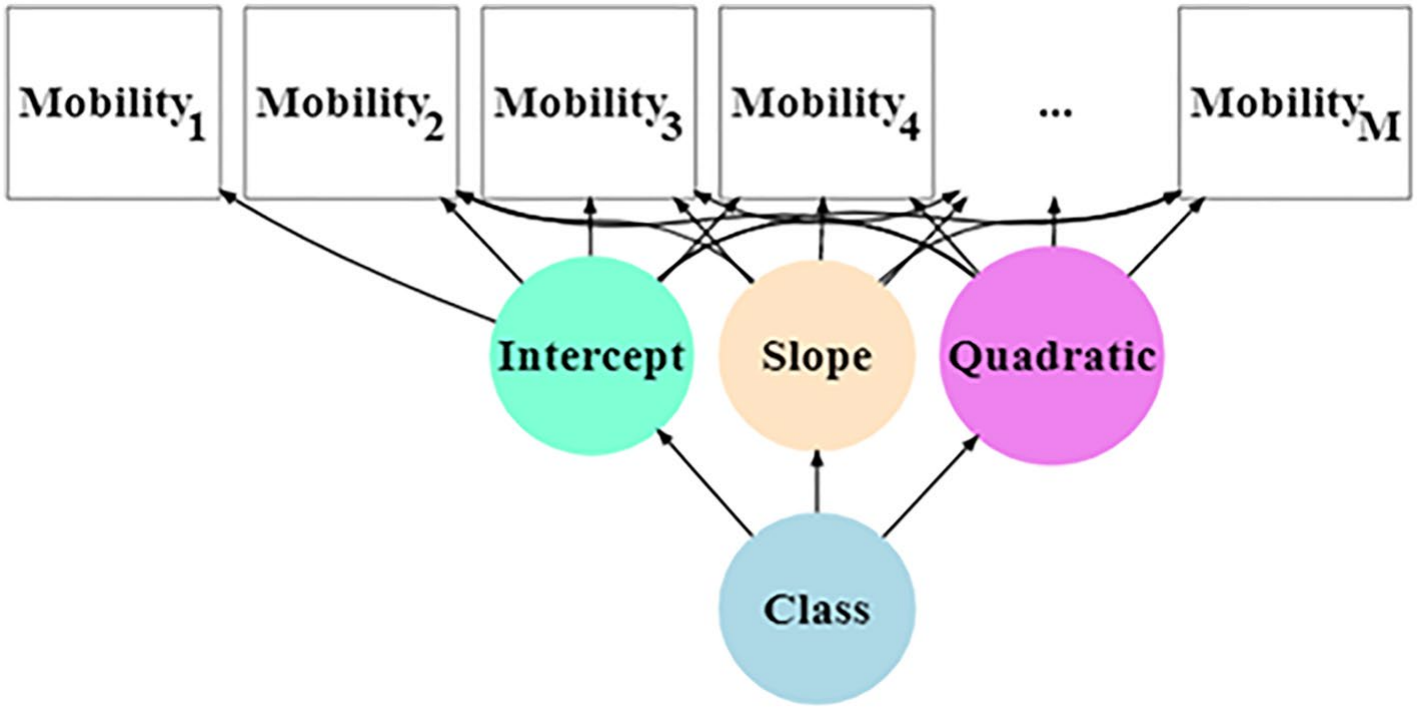
Mobility mixture model. Intercept and Mobility_1_ = baseline mobility; Slope = linear rate of change; Quadratic = quadratic rate of change; Class = categorical latent variable. Mobility_2_, …, Mobility_M_ are varying follow-up scores across 4.5 years for each subject.

**Figure 2:**
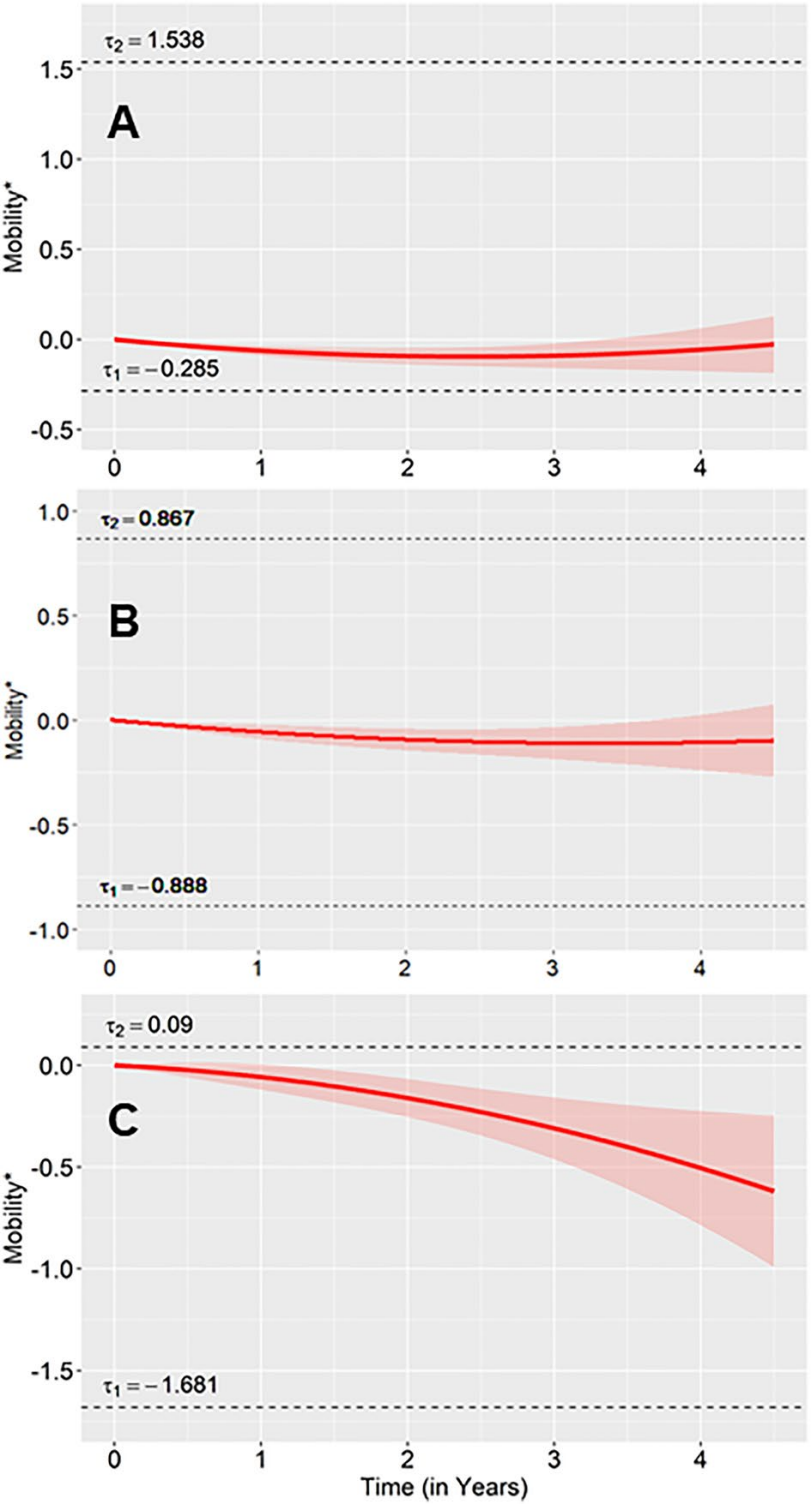
Quadratic regression of mobility. Shaded region represents a 95% confidence interval. The y-axis shows mobility* which is the normally distributed latent variable analytically inferred from the mobility ordered-categorical outcome. Thresholds (i.e. $\tau_{1}$ and $\tau_{2}$) are the values for mobility* for which the mobility ordered-categorical outcome crosses categories. Thus, a mobility* value above $\tau_{1}$ and below $\tau_{2}$ would signify that mobility is in response category one.

Descriptive statistics was completed for the entire sample and by disease duration strata. Kruskal–Wallis rank sum test and chi-square test assessed statistical significance in the comparison of continuous and categorical distributions across disease duration strata. LCGA allows for identifying meaningful clusters (or subgroups) within a larger study sample to examine longitudinal patterns over time^10–12^. We (1) performed LCGA to identify clusters of PWP based on longitudinal ambulatory impairment trajectories (see path diagram in Fig. 1), and (2) evaluated measures that may associate with cluster membership. The clusters, also termed *latent classes*, identified by LCGA are not known (observed) a priori but are determined empirically^10^. A trajectory shape for each class is estimated (i.e. intercept and slopes), and individuals can be assigned to the latent class of the highest probability of membership, which can be graphically displayed to facilitate interpretation^21^. A common approach for a LCGA of an ordered-categorical outcomes is to assume that a normally distributed latent variable exists from which each level of the observed categories is derived when the latent variable exceeds specific thresholds^10^. For analytical purposes, we inferred a latent variable mobility* with four thresholds based on the observed data of five categories. That is, for each PWP at each time point, the mobility PRO = 0 if the value of mobility* is less than the first threshold ($\tau_{1}$), the PRO = 1 if the value of mobility* is greater than the first threshold ($\tau_{1}$) but less than the second threshold ($\tau_{2}$), and so forth for increasing PRO responses (see Supplementary Methods for additional details). In the graphical displays, the threshold values for mobility* (which did not meaningfully vary over time) were denoted as $\tau_{1}$, $\tau_{2}$, $\tau_{3}$ and $\tau_{4}$.

Multivariable, multinomial logistic regression was used to determine if sociodemographic attributes influenced most likely cluster membership across strata (i.e. sociodemographic factors precede (lead to) cluster membership in a causal diagram). Odds ratios, 95% confidence intervals (CI) and p-values using z-tests for these multivariable models were reported. For other variables (military veteran status and clinical factors: depression, arthritis, anxiety, balance problems, pain, back pain duration and limitations, work in the past week, trouble getting out of bed, a car seat or a deep chair, freezing up, walking activities, light moderate and strenuous physical activities and muscle strength, OFF episodes, PD prescription medication), the directionality of the relationship with ambulatory impairment could not have been determined given the available data, therefore relationships between these variables and most likely cluster membership were evaluated using appropriate descriptive statistics (Kruskal–Wallis rank sum test or chi-square test) with a Bonferroni correction for multiple testing.

Statistical significance was defined by a two-tailed α = 0.05 (except when conducting the Bonferroni adjustment). LCGA was done using MPlus v8.6, and the MPlusAutomation package automated estimation and interpretation^22,23^. R program in the R studio environment was used for data management, graphical displays, and other statistical analyses.

## Results

### Descriptive analyses

The study population (Table 1) had an average age of 65.7 years (SD 9.5) and the majority (51%) were in the earliest stages of their disease (< 3 years from onset) at baseline. Forty six percent was female and 97% were white. A higher percentage of PWP had moderate to severe ambulatory impairment at baseline in those with longer PD disease duration. By disease duration strata, PWP did differ on most attributes, except for gender, OFF episodes, and light and moderate sport/recreational activities. The comparisons in Table 1 emphasizes that the study population differed by disease duration, therefore, underscoring the importance of modelling trajectories stratified by disease duration.

**Table 1:** Baseline characteristics of fox insight Parkinson’s disease study population *

	Overall		Disease duration						
	(N; %)		Early < 3 years		Mid 3–10 years		Later > 10 years		p^†^
N	16,863	100%	8612	51.1%	6181	36.7%	2070	12.3%	
EQ-5D-5L: Mobility impairment (%)									< 0.001
None	5683	33.7%	3622	42.1%	1754	28.4%	307	14.8%	
Slight	6902	40.9%	3495	40.6%	2636	42.7%	771	37.2%	
Moderate	3313	19.7%	1240	14.4%	1401	22.7%	672	32.5%	
Severe	873	5.2%	233	2.7%	357	5.8%	283	13.7%	
Not able to walk	86	0.5%	19	0.2%	30	0.5%	37	1.8%	
Sociodemographic attributes									
Body mass index (mean (SD))	26.55	(5.12)	26.74	(5.18)	26.39	(5.07)	26.22	(5.03)	< 0.001
Education (mean (SD))	4.8	(1.53)	4.82	(1.50)	4.82	(1.54)	4.72	(1.58)	0.032
Age (mean (SD))	65.74	(9.17)	64.7	(9.51)	66.68	(8.81)	67.28	(8.21)	< 0.001
Gender = female (%)	7577	(46.0)	3922	(46.5)	2740	(45.5)	915	(45.3)	0.400
Race = non-white (%)	439	(2.7)	205	(2.4)	182	(3.0)	52	(2.6)	0.091
Employment (%)									< 0.001
Full	3226	19.7%	2227	26.5%	867	14.5%	132	6.6%	< 0.001
Part-time	1307	8.0%	778	9.3%	426	7.1%	103	5.1%	< 0.001
Retired	11,055	67.5%	4986	59.4%	4400	73.5%	1669	83.2%	< 0.001
Unemployed	794	4.8%	398	4.7%	295	4.9%	101	5.0%	0.806
Clinical factors									
Veteran (%)	2434	14.8%	1218	14.5%	904	15.0%	312	15.5%	0.406
OFF episodes = Yes (%)	353	45.4%	140	33.9%	164	56.9%	49	63.6%	< 0.001
Current medication for PD = Yes (%)	14,717	90.4%	7009	83.8%	5780	97.2%	1928	97.7%	< 0.001
Current depression = Yes (%)	3725	25.5%	1880	25.3%	1318	24.7%	527	28.9%	0.001
Current anxiety = Yes (%)	4211	28.9%	2122	28.6%	1511	28.3%	578	31.8%	0.014
Current arthritis = Yes (%)	5938	40.7%	2971	40.0%	2172	40.8%	795	43.6%	0.018
Balance poor = Yes (%)	434	49.7%	282	45.5%	116	57.7%	36	67.9%	< 0.001
Current back pain = Yes (%)	5132	34.7%	2400	1.9%	1971	36.4%	761	41.0%	< 0.001
Back pain limit activities = Yes (%)	3633	70.8%	1637	68.2%	1421	72.1%	575	75.6%	< 0.001
Work-related activity = Yes (%)	6120	39.9%	3680	46.0%	1963	35.5%	477	26.2%	< 0.001
Pain (%)									< 0.001
None	4646	27.6%	2737	31.8%	1505	24.4%	404	19.6%	
Slight	7082	42.0%	3713	43.1%	2565	41.6%	804	39.0%	
Moderate	4270	25.4%	1855	21.6%	1736	28.1%	679	32.9%	
Severe	752	4.5%	275	3.2%	324	5.3%	153	7.4%	
Extreme	92	0.5%	27	0.3%	41	0.7%	24	1.2%	
Trouble getting out of bed, a care, or a deep chair (%)									< 0.001
Normal	5037	33.0%	3292	41.2%	1501	27.4%	244	13.4%	
Slight	6821	44.7%	3488	43.7%	2575	47.1%	758	41.8%	
Mild	2278	14.9%	904	11.3%	913	16.7%	461	25.4%	
Moderate	939	6.1%	257	3.2%	398	7.3%	284	15.6%	
Severe	199	1.3%	46	0.6%	85	1.6%	68	3.7%	
Problems with balance and walking									< 0.001
Normal	5206	34.1%	3459	43.3%	1514	27.7%	233	12.8%	
Slight	6639	43.5%	3425	42.9%	2541	46.4%	673	37.1%	
Mild	1603	10.5%	561	7.0%	671	12.3%	371	20.4%	
Moderate	1563	10.2%	488	6.1%	644	11.8%	431	23.7%	
Severe	263	1.7%	54	0.7%	102	1.9%	107	5.9%	
Suddenly stop or freeze when walking									< 0.001
Normal	11,177	73.2%	6656	83.3%	3760	68.7%	761	41.9%	
Slight	2329	15.2%	903	11.3%	981	17.9%	445	24.5%	
Mild	902	5.9%	256	3.2%	394	7.2%	252	13.9%	
Moderate	643	4.2%	128	1.6%	245	4.5%	270	14.9%	
Severe	223	1.5%	44	0.6%	92	1.7%	87	4.8%	
Walking activities									< 0.001
Never	1270	8.3%	590	7.4%	479	8.7%	201	11.0%	
Seldom	3074	20.0%	1501	18.7%	1126	20.4%	447	24.5%	
Sometimes	3899	25.4%	1985	24.8%	1428	25.8%	486	26.6%	
Often	7125	46.4%	3938	49.1%	2493	45.1%	694	38.0%	
Light sport and recreational activities									0.237
Never	9771	63.7%	5162	64.5%	3439	62.4%	1170	64.1%	
Seldom	2913	19.0%	1464	18.3%	1098	19.9%	351	19.2%	
Sometimes	1814	11.8%	947	11.8%	661	12.0%	206	11.3%	
Often	836	5.5%	429	5.4%	310	5.6%	97	5.3%	
Moderate sport and recreational activities									0.105
Never	11,547	75.4%	5965	74.8%	4161	75.5%	1421	77.9%	
Seldom	1916	12.5%	1004	12.6%	697	12.6%	215	11.8%	
Sometimes	1313	8.6%	711	8.9%	461	8.4%	141	7.7%	
Often	529	3.5%	290	3.6%	192	3.5%	47	2.6%	
Strenuous sport and recreational activities									< 0.001
Never	9538	62.3%	4690	58.8%	3571	64.8%	1277	70.1%	
Seldom	2067	13.5%	1120	14.0%	731	13.3%	216	11.9%	
Sometimes	2293	15.0%	1328	16.6%	737	13.4%	228	12.5%	
Often	1411	9.2%	842	10.6%	468	8.5%	101	5.5%	
Muscle strength									< 0.001
Never	5414	35.3%	2728	34.1%	1958	35.5%	728	39.8%	
Seldom	3907	25.5%	1988	24.9%	1464	26.5%	455	24.9%	
Sometimes	4130	26.9%	2203	27.5%	1478	26.8%	449	24.6%	
Often	1888	12.3%	1078	13.5%	615	11.2%	195	10.7%	

Mean ± standard deviation for continuous measures and number of subjects in each category for discrete measures with p-values reported from Kushall–Wallis and chi-square tests where appropriate.

^†^p < 0.05 is considered statistically significant.

### Average trajectory using the single cluster solution

When considering only a single cluster solution (the overall average trajectory), PWP had on average reported having slight ambulatory problems (starting above the first threshold $\tau_{1}$ but below the second threshold $\tau_{2}$ which corresponds to moderate problems) in each duration strata (Fig. 2). In the later disease duration stratum, the trajectory was closer to the $\tau_{2}$; thus, PWP in this stratum had higher ambulatory impairment on average. These single solution trajectories did not change substantially over time in review of the confidence intervals, though in the early disease stratum there was a small negative linear (Estimate = − 0.078, Standard Error [SE] = 0.021, p < 0.001) and positive quadratic effect (Estimate = 0.016, SE = 0.007, p = 0.02); in the mid-disease stratum there was a small negative linear effect (Estimate = − 0.067, SE = 0.024, p = 0.005); and there were no significant slope effects in the late disease stratum.

### Number of clusters by disease duration

Using LCGA, four latent classes best described perceived longitudinal ambulatory impairment patterns across the early and mid-disease duration strata, while five latent classes best described impairment in the later disease duration stratum. Across models, these solutions achieved a near minimum (< 1% decrease after in adding an additional class) for BIC, aBIC, AIC and AICC values (Supplementary Table 1) and were a near maximum entropy. Similarly, the interpretability of the classes supported these solutions across each of the stratified models.

### Description of clusters (subgroups)

**Figure 3:**
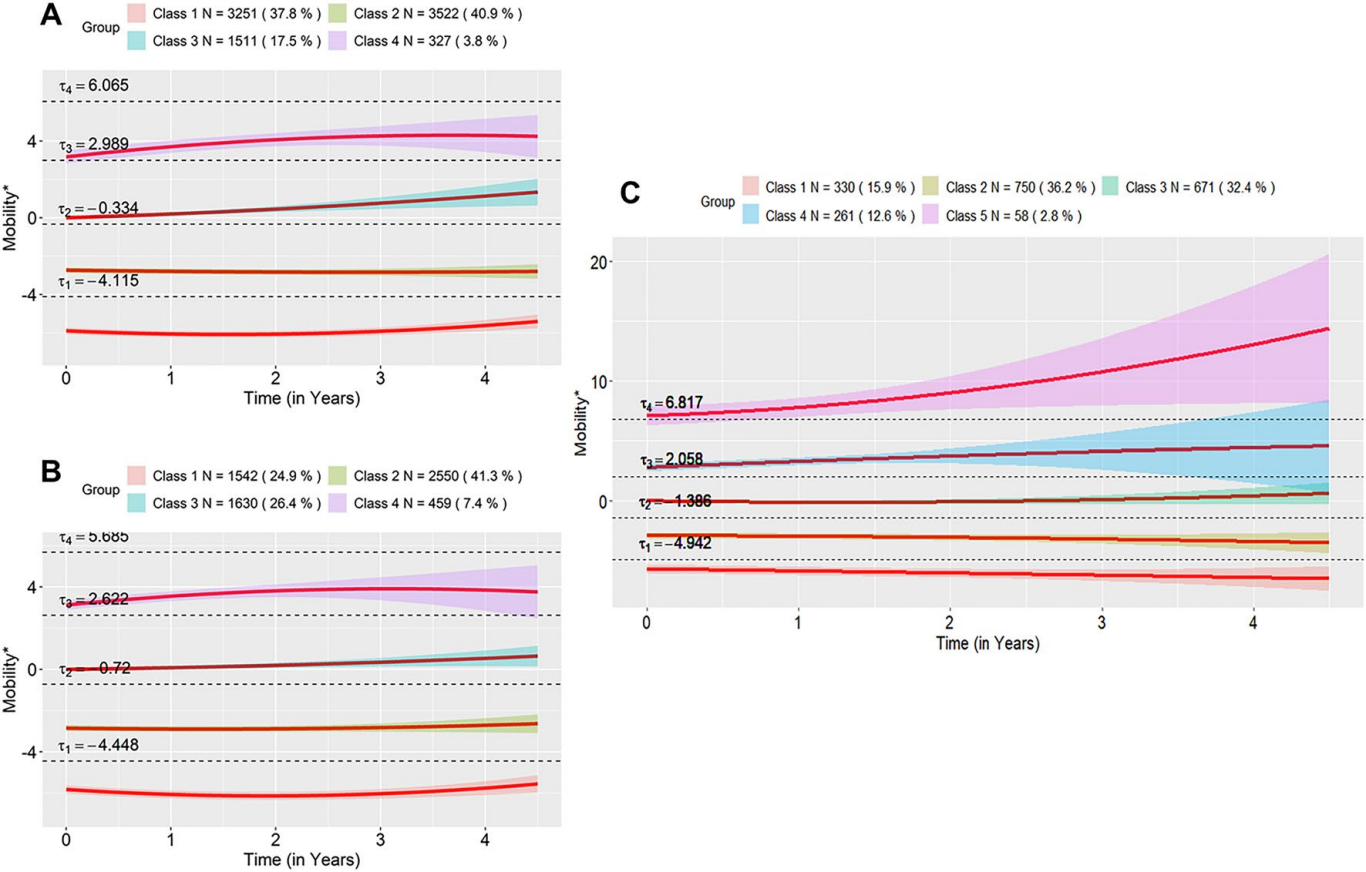
Average within-class trajectories across disease duration using quadratic regression. Shaded region in each plot represents a 95% Confidence Interval. The y-axis shows mobility* which is the normally distributed latent variable analytically inferred from the mobility ordered-categorical outcome. Thresholds (i.e. $\tau_{1}$, $\tau_{2}$, $\tau_{3}$ and $\tau_{4}$) are the values for mobility* for which the mobility ordered-categorical outcome crosses categories. Thus, the mobility response is one if the value of mobility* is greater than the first threshold, but less than the second threshold, the mobility response is two if the value of mobility* is greater than the second threshold, but less than the third threshold, the mobility response is three if the value of mobility* is greater than the third threshold, but less than the fourth threshold and the mobility response is four if the value of mobility* is greater than the fourth threshold.

The average ambulatory trajectories for each cluster within each disease duration strata are displayed in Fig. 3. In the early disease stratum, we labeled the four clusters as: no ambulatory impairment (Class 1: 37.8% [of participants]), slight impairment (Class 2: 40.7%), moderate impairment (Class 3: 17.5%) and severe impairment with variability (Class 4: 3.8%) (Fig. 3A). In the mid disease stratum, four subgroups were similarly described (Fig. 3B). In the later disease (> 10 years) stratum, we labeled Class 1 through Class 4 similarly, with Class 5 (2.8%) labeled as extreme impairment with variability as it was above $\tau_{4}$(Fig. 3C). The percentage of subjects in the moderate and severe subgroups increased with disease duration (Fig. 3). In contrast to the single cluster solutions in Fig. 2, that exhibit no change to slight improvement across strata, upon inspection of the individual trajectories per strata in Fig. 3, the slight to moderate impairment trajectories (Classes 1 and 2) are stable with time, will those in the moderate to extreme impairment trajectories (Classes 3 to 5) continue to accrue impairment with time—this emphasizes the importance of examining ambulation in the distinct cluster/subgroups of PWP rather than in the overall study population as an average trajectory.

### Cluster membership characteristics

Results from the multivariable multinomial logistic regression models with the least impaired cluster (Class 1) as the reference are presented in Table 2. Females were less likely to be in clusters with greater ambulatory impairment in PWP with mid-disease, but trending but mostly non-significant relationships in the other strata. On average, older age, higher BMI, lower education, lower income, and being unemployed versus retired were largely associated with increased assignment to clusters with high impairment across disease duration strata. Also, those employed had less impairment compared to retirees. There was also no evidence to suggest differences between white and non-white PWP in cluster membership (although there is an imbalance in the distribution by race in the study population—see Table 1).

**Table 2:** Multinomial regression results for each disease duration strata with the least impaired (Class 1) cluster as the reference category.

Attribute	Early < 3 years		Mid 3–10 years		Later > 10 years	
	Odds ratio (95% CI)	p*	Odds ratio (95% CI)	p*	Odds ratio (95% CI)	p*
Class 2 vs Class 1						
Gender = female	0.93 (0.83, 1.04)	0.2	0.83 (0.71, 0.96)	**0.012**	0.86 (0.64, 1.16)	0.3
BMI	1.07 (1.06, 1.08)	** < 0.001**	1.05 (1.03, 1.07)	** < 0.001**	1.07 (1.04, 1.11)	** < 0.001**
Race = non-white	1.06 (0.74, 1.52)	0.8	1.6 (1.02, 2.50)	**0.04**	0.72 (0.31, 1.64)	0.4
Education	0.95 (0.92, 0.99)	**0.017**	0.99 (0.94, 1.04)	0.7	0.97 (0.88, 1.07)	0.6
Income	0.87 (0.84, 0.91)	** < 0.001**	0.9 (0.86, 0.95)	** < 0.001**	0.89 (0.80, 0.99)	**0.025**
Full employment vs retired	0.94 (0.81, 1.09)	0.4	1.04 (0.84, 1.30)	0.7	0.78 (0.47, 1.28)	0.3
Part employment vs retired	0.97 (0.80, 1.17)	0.8	1.16 (0.88, 1.53)	0.3	0.81 (0.45, 1.47)	0.5
Unemployment vs retired	1.56 (1.15, 2.11)	**0.004**	2.11 (1.41, 3.16)	** < 0.001**	0.99 (0.45, 2.16)	> 0.9
Age	1 (1.00, 1.01)	0.3	1 (0.99, 1.01)	0.4	0.99 (0.97, 1.01)	0.3
Class 3 vs Class 1						
Gender = female	0.86 (0.75, 1.00)	**0.047**	0.76 (0.65, 0.90)	**0.001**	0.79 (0.58, 1.07)	0.13
BMI	1.12 (1.11, 1.14)	** < 0.001**	1.12 (1.10, 1.14)	** < 0.001**	1.09 (1.06, 1.13)	** < 0.001**
Race = non-white	1.35 (0.85, 2.15)	0.2	1.49 (0.89, 2.48)	0.13	0.48 (0.18, 1.22)	0.12
Education	0.91 (0.87, 0.96)	** < 0.001**	0.96 (0.91, 1.02)	0.2	0.99 (0.89, 1.09)	0.8
Income	0.78 (0.74, 0.82)	** < 0.001**	0.8 (0.76, 0.85)	** < 0.001**	0.8 (0.72, 0.89)	** < 0.001**
Full employment vs retired	0.69 (0.56, 0.85)	** < 0.001**	0.72 (0.55, 0.94)	**0.017**	0.48 (0.27, 0.86)	**0.013**
Part employment vs retired	0.71 (0.54, 0.92)	**0.011**	0.94 (0.68, 1.31)	0.7	0.71 (0.38, 1.34)	0.3
Unemployment vs retired	1.85 (1.30, 2.65)	** < 0.001**	1.86 (1.19, 2.93)	**0.007**	1.58 (0.73, 3.43)	0.2
Age	1.02 (1.01, 1.03)	** < 0.001**	1.02 (1.01, 1.04)	** < 0.001**	1.02 (1.00, 1.04)	0.13
Class 4 vs Class 1						
Gender = female	0.95 (0.73, 1.23)	0.7	0.71 (0.55, 0.91)	**0.007**	0.57 (0.39, 0.85)	**0.005**
BMI	1.15 (1.13, 1.18)	** < 0.001**	1.14 (1.11, 1.17)	** < 0.001**	1.12 (1.07, 1.16)	** < 0.001**
Race = non-white	1.44 (0.59, 3.51)	0.4	1.83 (0.90, 3.76)	0.1	1.11 (0.39, 3.20)	0.8
Education	0.84 (0.77, 0.91)	** < 0.001**	0.93 (0.85, 1.01)	0.066	1.04 (0.92, 1.19)	0.5
Income	0.74 (0.68, 0.81)	** < 0.001**	0.71 (0.66, 0.77)	** < 0.001**	0.69 (0.60, 0.79)	** < 0.001**
Full employment vs retired	0.37 (0.23, 0.62)	** < 0.001**	0.55 (0.32, 0.94)	**0.029**	0.11 (0.02, 0.46)	**0.003**
Part employment vs retired	0.32 (0.16, 0.64)	**0.001**	0.48 (0.24, 0.98)	**0.043**	0.23 (0.06, 0.80)	**0.021**
Unemployment vs retired	3.06 (1.78, 5.26)	** < 0.001**	3.86 (2.14, 6.97)	** < 0.001**	3.17 (1.33, 7.57)	**0.009**
Age	1.07 (1.05, 1.09)	** < 0.001**	1.07 (1.06, 1.09)	** < 0.001**	1.05 (1.02, 1.07)	** < 0.001**
Class 5 vs Class 1						
Gender = female	No fifth cluster		No fifth cluster		0.88 (0.46, 1.66)	0.7
BMI					1.13 (1.06, 1.20)	** < 0.001**
Race = non-white					2.18 (0.43, 11.1)	0.3
Education					0.83 (0.67, 1.03)	0.087
Income					0.88 (0.70, 1.10)	0.3
Full employment vs retired					0 (0.00, 0.00)	** < 0.001**
Part employment vs retired					0.72 (0.16, 3.29)	0.7
Unemployment vs retired					2.05 (0.39, 10.7)	0.4
Age					1.1 (1.05, 1.14)	** < 0.001**

* Bolded p-values are statistically significant at the two-sided alpha threshold of 5%.

Descriptive statistics are reported for sociodemographic and clinical variables in Supplementary Tables 2–4. In brief, in the early disease stratum, the higher impairment classes include a higher percentage of PWP on prescribed PD medications, with a greater prevalence of depression, anxiety and arthritis. The higher classes also reported more impairment in balance, back pain problems, walking impairment, pain and trouble getting out of bed and less work-related activity, sport and recreational activities (light, moderate and strenuous) and muscle strength. Class 3 had a higher proportion of PWP with current depression and anxiety than Class 4, while Class 4 had more physical impairment than Class 3. These trends were similar in the mid disease stratum, except Class 4 had higher percentages of current depression and anxiety than Class 3. There were also no differences in the percentage on PD medication (given the Bonferroni correction). The later duration stratum continued similar trends as the mid disease stratum, except there were no differences in the proportion of veterans or PWP with balance impairment across clusters.

## Discussion

Ambulatory impairment is common in PWP, with a heterogenous presentation that negatively impacts QoL^1,2^. Little is known about how PWP experience their difficulty in walking, much less over time, and by disease duration. Studies that have analyzed ambulation in PWP have done so in aggregate, and resultantly fail to observe intrinsic and meaningful variation in subgroup ambulatory patterns—which is highly relevant for PROs. The analysis of subgroup mobility PRO trajectories in PWP is essential for a holistic understanding the progression of ambulatory impairment. Here we leveraged a readily accessible and broadly used health-related QoL instrument to identify and characterize subgroups of PWP with similar perceived ambulatory impairment trajectories over time and stratified by disease duration. Consistent with prior research^24^, a higher percentage of PWP had moderate to severe ambulatory impairment at baseline in those with longer PD disease duration. PWP at the early and mid-disease stages of PD were clustered into four trajectories with > 65% having no to slight and stable impairment, and > 20% having moderate to severe trajectories that were increasing over time. PWP at the later stage of PD were clustered into five trajectories, including 2.8% in an extremely impaired subgroup—in general, ~ 50% had at modest slight and stable impairment while the other ~ 50% had moderate to extreme impairment that increased with time. There were also significant associations with trajectory membership for multiple sociodemographic and clinical attributes, which offers insights to drivers and correlates of heterogeneity in ambulatory impairment. Collectively, the findings may be leveraged to identify PWP at risk for greater sustained ambulatory impairment and may be utilized in patient-centered care approaches to advance care management and shared decision making.

The multivariable models provided new insights into ambulatory impairment in PWP. For example, despite comparing multiple facets of PD presentation, it has been unclear to extent to which there may be gender differences in motor functioning, mobility, and health-related quality of life^25,26^. As evident from the multinomial models where we adjusted for likely confounders, we observed females were less like to be in the more impaired clusters in those with mid-disease. There were an underrepresentation of females in Class 3 vs Class 1 during the earliest stage of PD and Class 4 vs Class 1 during the later stage of PD, which highlights that there is a non-linear relationship between sex and walking difficulties over the disease course—which, in part, may explain the unclear patterns previously observed by others^25,26^. The relationships for employed were as one would speculate, with part/full-time employed PWP being less burdened with high impairment compared to retirees across disease duration strata, while unemployed PWP (which would include those on disability) were more much likely to be in clusters with more severe impairment compared to retirees. Lower income was consistently associated with higher impairment and consistent with prior findings^27^. This effect was irrespective of disease course which illustrated how profound social inequities can impact PD outcomes. Another social determinant of health, higher education, has been inversed associated with white matter hyperintensities and lower MDS-UPDRS scores independent of nigrostriatal dopaminergic denervation in PWP^28^. Here, we observed higher education having a protective effect in relation to perceived ambulatory impairment only during at the earliest stage of PD, and merits further investigation into the relationship of resilience and PD progression. Another key observation that requires further inquiry, are the patterns observed for race. We did not observe substantial differences in longitudinal ambulatory impairment between white and non-white PWP when not adjusting and when adjusting for other social determinants of health. This lack of a longitudinal difference is intriguing considering cross-sectional racial difference observed for other health-related quality of life measures^29^. Our observation may be driven by the modest non-white subset in the current data, or that we were able to adjust for key socioeconomic variables (i.e. education, employment, and income)—others have observed that adjusting for income and education mitigated racial differences in PD severity models^27^. Thus, considering socioeconomic conditions are downstream of race in a causal diagram, subsequent work should explore causal mediation analyses to determine the extent to which social inequities drive racial differences in PD. In our post-hoc analyses, we noted that PWP with poorer mental health, higher burden of pain, and being a veteran were associated with a higher burden of ambulatory impairment—this may inform care conversations related to PD management and prognostication by aiding efforts to identify PWP most vulnerable for long-term adverse outcomes in functional mobility.

These findings offer new perspectives on the longitudinal ambulatory experiences of PWP, from a person-centered framework. Understanding the anticipated trajectories PWP will experience will facilitate the development of tailored care/treatment strategies and allow for greater allocation of resources particularly for those with sudden increases in impairment and for those with moderate to extreme impairment that does not decrease with time. The findings also have great potential for developing novel endpoints for clinical and observational research. It would also be important to determine the underlying symptomatology for individual clusters and the extent to which these symptoms are preventable, treatable, or l-3,4-dihydroxyphenylalanine (Levodopa) responsive. It would also be information to focus on PWP who ambulatory impairment remained low and explore what risk and care strategies may have contributed to these favorable trends. Lastly, more granular baseline data such as subdivision of PWP into heterogeneous PD subtypes (i.e. tremor-dominant versus PIGD) and incorporating genetic and biomarker data may allow better prediction of walking trajectories as experienced by PWP.

There are several strengths in the current study, including the large sample size, the application of LCGA to discern subgroups, the opportunity to stratify models by disease duration, the availability of longitudinal EQ-5D-5L data, and the extensive baseline information. There are a few limitations to acknowledge, the first is the study population was comprised of PWP who were digitally literate and therefore it may not represent the cognitively impaired or other marginalized subpopulations. There was also an underrepresentation of Non-White PWP in the data, therefore these results might have limited generalizability to Non-White populations. However, PWP in Fox Insight are comparable to PWP who participated in-person cohort studies, with similar a burden of difficulties in walking^30^. But we do acknowledge that there is an absence of potentially informative measures, including clinician-rated measures of PD severity, details on healthcare provider team, and treatment availability/access. This study also assumes that all LCGA model assumptions were met in this PD sample for valid inference under the special considerations in which the latent variable mobility* was used as the outcome^31^. We did perform more robust inference in case there is a violation of model parametric assumptions and included quadratic terms in our models in case the trajectory of ambulatory impairment is non-linear. A key limitation is that in our chosen solutions, there were some clusters of a small cell size, and the entropy values and a few of the posterior probability of membership averages were < 70%. A final limitation is the PRO used is a single-item measure that focused on walking difficulties; thus, multi-item mobility PROs and objective measures (e.g. timed performance tasks or real-world data from wearable devices) will allow for create resolution and a more holistic understand of ambulation in PWP.

In summary, LCGA uncovered multiple distinct ambulatory impairment trajectories and distinct subgroups of PWP based on their experiences with difficulties in walking. This is consistent with our prior work on pain perceptions, emphasizing the need the account for longitudinal heterogeneity in PD symptomatology, the need to factor in disease duration, and the power of PRO for facilitating these discoveries^12^. We hope that this work can serve as a framework for characterizing other complex PD impairments, as well as impairment in other chronic disorders, which may subsequently optimize patient care and facilitate the discovery of modifiable risk factors for symptom exacerbation by serving as robust phenotypes for clinical and observational research.

### Supplementary Information

Supplementary Information.

Supplementary Tables.

## Data Availability

The Fox Insight Study data are available to others through the Fox DEN (https://foxden.michaeljfox.org/). The data used in this study is available from the authors to qualified researchers with Fox Insight Data Use approval (https://foxden.michaeljfox.org/insight/register/). Please contact the corresponding author for more information.
