# Supplementary material for: A person-centered approach to characterizing longitudinal ambulatory impairment in Parkinson's disease
Source: Sci Rep. 2024 May 20;14:11509. doi: 10.1038/s41598-024-62179-9 (PMC11106289; doi:10.1038/s41598-024-62179-9)
Supplement: Supplementary file 1 — Supplementary Information. [file 41598_2024_62179_MOESM1_ESM.docx]

**Supplementary Methods**

LCGA Modelling

For these LCGA a quadratic term was included to allow for the possibility of a non-linear trajectory of change over time and data was stratified by disease duration (early, mid, and later). In line with this latent variable method for estimating mobility*, the interpretation of the model slopes, linear and quadratic, respectively, are the change in the z-score of a normal distribution for a one-unit change in time (years) and time squared.

We used the MLR option in MPlus to perform model estimation ^1, 2^, which uses maximum likelihood to estimate the parameters and a robust Huber-White sandwich estimator to calculate standard errors ^2^. Robust methods are insensitive to departures from normality assumptions about the data. The approach in MPlus effectively handled ignorable missing data dependent on the data in hand (i.e., “missing at random” assumption) via full information maximum likelihood. As a result, participants with missing data were included in the trajectory analysis for unbiased inference.

To identify the optimal number of classes, statistical indices and parameter estimates are evaluated, including entropy, Akaike information criterion (AIC), Bayesian information criterion (BIC), sample-size adjusted BIC (aBIC), corrected AIC for small sample sizes (AICC) and assessment of the number of subjects assigned into each class ^3^. The first solution evaluated is a single cluster solution (i.e. model of the mean intercept and slopes across the entire sample), to which solutions of increasing the number of clusters (e.g. 2, 3, 4, 5, 6, 7 and 8) are compared until the best solution is identified.

More generally, in LCGA, within each cluster the intercept and slopes are latent variables and the clustering variable is a latent variable. Most likely cluster membership for each individual in the study sample can be summarized in an observed nominal variable by categorizing each individual into the cluster of the highest probability of membership. For example, hypothetically in a two cluster model, if an individual is a member of cluster A with probability 0.80 and cluster B with probability 0.20, they are most likely a member of cluster A.

1. Muthén LK, Muthén BO. Mplus. The comprehensive modelling program for applied researchers: user’s guide 2012;5.

2. Huber PJ. The behavior of maximum likelihood estimates under nonstandard conditions. Proceedings of the fifth Berkeley symposium on mathematical statistics and probability; 1967. p. 221-233.

3. Schwarz G. Estimating the dimension of a model. The annals of statistics 1978;6(2):461-464.
