# Supplementary material for: A person-centered approach to characterizing longitudinal ambulatory impairment in Parkinson's disease
Source: Sci Rep. 2024 May 20;14:11509. doi: 10.1038/s41598-024-62179-9 (PMC11106289; doi:10.1038/s41598-024-62179-9)
Supplement: Supplementary file 2 — Supplementary Tables. [file 41598_2024_62179_MOESM2_ESM.docx]

**Supplementary Table 1.** Model selection measures for LCGA of mobility problems in people with Parkinson’s disease stratified by disease duration

| **Number of Classes** | **AIC** | **BIC** | **aBIC** | **AICC** | **Entropy** |
| --- | --- | --- | --- | --- | --- |
| **Disease Duration < 3 years** | | | | | |
| **1** | 80491.76 | 80534.12 | 80515.06 | 80491.77 |  |
| **2** | 67526.20 | 67596.80 | 67526.22 | 67565.02 | 0.799 |
| **3** | 63133.10 | 63231.95 | 63133.14 | 63187.46 | 0.790 |
| **4** | 62176.48 | 62303.57 | 62176.56 | 62246.37 | 0.786 |
| **5** | 61811.46 | 61966.80 | 61811.58 | 61896.89 | 0.687 |
| **6** | 61613.12 | 61796.70 | 61613.28 | 61714.08 | 0.645 |
| **Disease Duration 3-10 years** | | | | | |
| **1** | 64477.00 | 64517.38 | 64498.31 | 64477.02 |  |
| **2** | 54850.60 | 54917.89 | 54850.63 | 54886.11 | 0.782 |
| **3** | 51069.37 | 51163.58 | 51069.44 | 51119.09 | 0.785 |
| **4** | 49816.22 | 49937.35 | 49816.33 | 49880.15 | 0.769 |
| **5** | 49516.26 | 49664.30 | 49516.42 | 49594.39 | 0.786 |
| **6** | 49259.04 | 49434.00 | 49259.27 | 49351.38 | 0.704 |
| **Disease Duration >10 years** | | | | | |
| **1** | 21578.48 | 21612.29 | 21593.23 | 21578.52 |  |
| **2** | 18516.37 | 18572.73 | 18516.48 | 18540.96 | 0.810 |
| **3** | 17444.71 | 17523.60 | 17444.91 | 17479.12 | 0.789 |
| **4** | 16898.94 | 17000.38 | 16899.27 | 16943.19 | 0.761 |
| **5** | 16697.66 | 16821.63 | 16698.15 | 16751.74 | 0.773 |
| **6** | 16641.28 | 16787.80 | 16641.97 | 16705.19 | 0.699 |

AIC=Akaike Information Criterion; BIC=Bayesian Information Criterion; aBIC=Sample-Size Adjusted BIC; AICC=Corrected AIC for Small Sample Sizes

**Supplementary Table 2.** Post-hoc analyses of baseline sociodemographic and clinical factors at baseline across most likely cluster assignment for early disease duration < 3 years.

|  | Most likely cluster assignment | | | | | | | |  |
| --- | --- | --- | --- | --- | --- | --- | --- | --- | --- |
|  | 1 |  | 2 |  | 3 |  | 4 |  | p |
| n | 3251 |  | 3522 |  | 1511 |  | 327 |  |  |
| Mobility Impairment (%) |  |  |  |  |  |  |  |  | <0.001 |
| None | 2895 | (89.1) | 701 | (19.9) | 25 | (1.7) | 1 | (0.3) |  |
| Slight | 350 | (10.8) | 2578 | (73.2) | 559 | (37.0) | 8 | (2.4) |  |
| Moderate | 4 | (0.1) | 231 | (6.6) | 879 | (58.2) | 126 | (38.5) |  |
| Severe | 0 | 0.0 | 11 | (0.3) | 48 | (3.2) | 174 | (53.2) |  |
| Extreme | 0 | 0.0 | 1 | 0.0 | 0 | 0.0 | 18 | (5.5) |  |
|  |  |  |  |  |  |  |  |  |  |
| Sociodemographics |  |  |  |  |  |  |  |  |  |
| BMI (mean (SD)) | 25.44 | (4.2) | 26.92 | (5.1) | 28.49 | (6.1) | 29.39 | (7.2) | <0.001 |
| Education (mean (SD)) | 5.01 | (1.4) | 4.79 | (1.5) | 4.56 | (1.6) | 4.35 | (1.7) | <0.001 |
| Income (mean (SD)) | 4.87 | (1.4) | 4.51 | (1.5) | 4.08 | (1.6) | 3.72 | (1.7) | <0.001 |
| Age (mean (SD)) | 64.04 | (8.9) | 64.27 | (9.5) | 66.08 | (10.2) | 69.45 | (9.8) | <0.001 |
| Sex = Female (%) | 1535 | (48.0) | 1567 | (45.6) | 663 | (45.0) | 156 | (48.4) | 0.125 |
| Race = Non-White (%) | 70 | (2.2) | 88 | (2.6) | 39 | (2.6) | 8 | (2.5) | 0.721 |
| Employment (%) |  |  |  |  |  |  |  |  | <0.001 |
| Full | 971 | (30.5) | 957 | (28.0) | 275 | (18.8) | 24 | (7.5) | <0.001 |
| Part-time | 336 | (10.6) | 328 | (9.6) | 104 | (7.1) | 10 | (3.1) | <0.001 |
| Retired | 1778 | (55.9) | 1966 | (57.5) | 983 | (67.2) | 258 | (80.6) | <0.001 |
| Unemployed | 98 | (3.1) | 171 | (5.0) | 101 | (6.9) | 28 | (8.8) | <0.001 |
|  |  |  |  |  |  |  |  |  |  |
| Clinical factors |  |  |  |  |  |  |  |  |  |
| Veteran (%) | 387 | (12.1) | 487 | (14.2) | 279 | (19.0) | 65 | (20.3) | <0.001 |
| OFF Episodes = Yes (%) | 45 | (28.7) | 56 | (33.1) | 35 | (46.1) | 4 | (36.4) | 0.072 |
| Current Medication for PD = Yes (%) | 2514 | (79.3) | 2882 | (84.1) | 1322 | (91.1) | 290 | (92.1) | <0.001 |
| Current Depression | 459 | (16.2) | 115 | (41.5) | 806 | (26.5) | 500 | (39.1) | <0.001 |
| Current Anxiety | 613 | (21.6) | 107 | (38.6) | 911 | (30.0) | 491 | (38.4) | <0.001 |
| Current Arthritis = Yes (%) | 851 | (30.0) | 1242 | (40.8) | 690 | (54.0) | 187 | (67.3) | <0.001 |
| Balance Poor = Yes (%) | 58 | (18.7) | 145 | (64.4) | 64 | (91.4) | 15 | (100.0) | <0.001 |
| Current Back Pain = Yes (%) | 580 | (20.3) | 1052 | (34.2) | 614 | (47.0) | 154 | (54.8) | <0.001 |
| Back Pain Limit Activities = Yes (%) | 289 | (49.8) | 712 | (67.7) | 506 | (82.4) | 130 | (84.4) | <0.001 |
| Work-related Activity = Yes (%) | 1618 | 52.8 | 1538 | 47.1 | 468 | 34.3 | 56 | 18.6 | <0.001 |
| Pain (%) |  |  |  |  |  |  |  |  | <0.001 |
| None | 1551 | (47.7) | 924 | (26.2) | 223 | (14.8) | 39 | (12.0) |  |
| Slight | 1368 | (42.1) | 1738 | (49.4) | 525 | (34.8) | 82 | (25.2) |  |
| Moderate | 313 | (9.6) | 779 | (22.1) | 619 | (41.0) | 144 | (44.2) |  |
| Severe | 18 | (0.6) | 77 | (2.2) | 132 | (8.7) | 47 | (14.4) |  |
| Extreme | 0 | 0.0 | 2 | (0.1) | 11 | (0.7) | 14 | (4.3) |  |
| Trouble Getting out of bed, a care, or a deep chair (%) |  |  |  |  |  |  |  |  | <0.001 |
| Normal | 2082 | (68.2) | 1058 | (32.3) | 141 | (10.4) | 10 | (3.3) |  |
| Slight | 898 | (29.4) | 1814 | (55.5) | 684 | (50.3) | 92 | (30.6) |  |
| Mild | 71 | (2.3) | 349 | (10.7) | 388 | (28.6) | 96 | (31.9) |  |
| Moderate | 4 | (0.1) | 49 | (1.5) | 133 | (9.8) | 71 | (23.6) |  |
| Severe | 0 | 0.0 | 1 | 0.0 | 13 | (1.0) | 32 | (10.6) |  |
| Problems with Balance and Walking |  |  |  |  |  |  |  |  | <0.001 |
| Normal | 2461 | (80.6) | 920 | (28.1) | 77 | (5.7) | 1 | (0.3) |  |
| Slight | 575 | (18.8) | 2067 | (63.2) | 731 | (53.8) | 51 | (16.9) |  |
| Mild | 19 | (0.6) | 209 | (6.4) | 290 | (21.3) | 43 | (14.3) |  |
| Moderate | 0 | 0.0 | 71 | (2.2) | 251 | (18.5) | 166 | (55.1) |  |
| Severe | 0 | 0.0 | 4 | (0.1) | 10 | (0.7) | 40 | (13.3) |  |
| Suddenly stop or freeze when walking |  |  |  |  |  |  |  |  | <0.001 |
| Normal | 2986 | (97.7) | 2777 | (84.9) | 793 | (58.4) | 100 | (33.2) |  |
| Slight | 66 | (2.2) | 422 | (12.9) | 355 | (26.1) | 59 | (19.6) |  |
| Mild | 3 | (0.1) | 64 | (2.0) | 130 | (9.6) | 59 | (19.6) |  |
| Moderate | 0 | 0.0 | 8 | (0.2) | 73 | (5.4) | 47 | (15.6) |  |
| Severe | 0 | 0.0 | 0 | 0.0 | 8 | (0.6) | 36 | (12.0) |  |
| Walking Activities |  |  |  |  |  |  |  |  | <0.001 |
| Never | 131 | (4.3) | 209 | (6.4) | 140 | (10.2) | 110 | (36.4) |  |
| Seldom | 442 | (14.4) | 633 | (19.3) | 351 | (25.7) | 75 | (24.8) |  |
| Sometimes | 713 | (23.2) | 853 | (26.1) | 369 | (27.0) | 50 | (16.6) |  |
| Often | 1783 | (58.1) | 1579 | (48.2) | 508 | (37.1) | 67 | (22.2) |  |
| Light sport and recreational activities |  |  |  |  |  |  |  |  | <0.001 |
| Never | 1854 | (60.5) | 2061 | (63.0) | 984 | (72.1) | 262 | (86.8) |  |
| Seldom | 580 | (18.9) | 636 | (19.4) | 225 | (16.5) | 23 | (7.6) |  |
| Sometimes | 408 | (13.3) | 415 | (12.7) | 110 | (8.1) | 14 | (4.6) |  |
| Often | 221 | (7.2) | 159 | (4.9) | 46 | (3.4) | 3 | (1.0) |  |
| Moderate sport and recreational activities |  |  |  |  |  |  |  |  | <0.001 |
| Never | 2113 | (69.2) | 2414 | (74.1) | 1152 | (84.6) | 285 | (95.0) |  |
| Seldom | 458 | (15.0) | 434 | (13.3) | 105 | (7.7) | 7 | (2.3) |  |
| Sometimes | 330 | (10.8) | 304 | (9.3) | 71 | (5.2) | 6 | (2.0) |  |
| Often | 151 | (4.9) | 104 | (3.2) | 33 | (2.4) | 2 | (0.7) |  |
| Strenuous sport and recreational activities |  |  |  |  |  |  |  |  | <0.001 |
| Never | 1462 | (47.8) | 1976 | (60.6) | 996 | (73.2) | 256 | (85.6) |  |
| Seldom | 483 | (15.8) | 466 | (14.3) | 152 | (11.2) | 19 | (6.4) |  |
| Sometimes | 630 | (20.6) | 538 | (16.5) | 144 | (10.6) | 16 | (5.4) |  |
| Often | 486 | (15.9) | 280 | (8.6) | 68 | (5.0) | 8 | (2.7) |  |
| Muscle strength |  |  |  |  |  |  |  |  | <0.001 |
| Never | 920 | (30.0) | 1134 | (34.7) | 554 | (40.6) | 120 | (40.0) |  |
| Seldom | 745 | (24.3) | 853 | (26.1) | 322 | (23.6) | 68 | (22.7) |  |
| Sometimes | 948 | (30.9) | 850 | (26.0) | 325 | (23.8) | 79 | (26.3) |  |
| Often | 451 | (14.7) | 429 | (13.1) | 165 | (12.1) | 33 | (11.0) |  |

**Supplementary Table 3.** Post-hoc analyses of baseline sociodemographic and clinical factors at baseline across most likely cluster assignment for mid disease duration 3-10 years.

|  | Most likely cluster assignment | | | | | | | |  |
| --- | --- | --- | --- | --- | --- | --- | --- | --- | --- |
|  | 1 |  | 2 |  | 3 |  | 4 |  | p |
| n | 1542 |  | 2550 |  | 1629 |  | 459 |  |  |
| Mobility Impairment (%) |  |  |  |  |  |  |  |  | <0.001 |
| None | 1285 | (83.3) | 453 | (17.8) | 15 | (0.9) | 1 | (0.2) |  |
| Slight | 252 | (16.3) | 1916 | (75.2) | 461 | (28.3) | 6 | (1.3) |  |
| Moderate | 3 | (0.2) | 175 | (6.9) | 1072 | (65.9) | 151 | (32.9) |  |
| Severe | 1 | (0.1) | 4 | (0.2) | 78 | (4.8) | 274 | (59.7) |  |
| Extreme | 1 | (0.1) | 1 | 0.0 | 1 | (0.1) | 27 | (5.9) |  |
|  |  |  |  |  |  |  |  |  |  |
| Sociodemographics |  |  |  |  |  |  |  |  |  |
| BMI (mean (SD)) | 24.99 | (4.0) | 26.15 | (4.7) | 27.49 | (5.7) | 28.06 | (6.8) | <0.001 |
| Education (mean (SD)) | 4.95 | (1.5) | 4.85 | (1.5) | 4.71 | (1.6) | 4.54 | (1.7) | <0.001 |
| Income (mean (SD)) | 4.69 | (1.5) | 4.44 | (1.5) | 4.06 | (1.6) | 3.64 | (1.7) | <0.001 |
| Age (mean (SD)) | 66.01 | (7.6) | 65.78 | (8.8) | 67.55 | (9.2) | 70.82 | (9.6) | <0.001 |
| Sex = Female (%) | 734 | (48.5) | 1118 | (44.9) | 696 | (44.2) | 192 | (43.0) | 0.047 |
| Race = Non-White (%) | 39 | (2.6) | 88 | (3.5) | 42 | (2.7) | 13 | (2.9) | 0.264 |
| Employment (%) |  |  |  |  |  |  |  |  |  |
| Full | 266 | (17.6) | 421 | (17.0) | 160 | (10.2) | 20 | (4.5) | <0.001 |
| Part-time | 120 | (8.0) | 200 | (8.1) | 96 | (6.1) | 10 | (2.3) | <0.001 |
| Retired | 1079 | (71.5) | 1708 | (69.0) | 1228 | (78.7) | 384 | (86.7) | <0.001 |
| Unemployed | 44 | (2.9) | 145 | (5.9) | 77 | (4.9) | 29 | (6.5) | <0.001 |
|  |  |  |  |  |  |  |  |  |  |
| Clinical factors |  |  |  |  |  |  |  |  |  |
| Veteran (%) | 185 | (12.2) | 345 | (13.9) | 284 | (18.1) | 90 | (20.3) | <0.001 |
| OFF Episodes = Yes (%) | 34 | (39.5) | 58 | (54.2) | 57 | (75.0) | 15 | (78.9) | <0.001 |
| Current Medication for PD = Yes (%) | 1444 | (96.7) | 2405 | (97.8) | 1517 | (97.2) | 414 | (95.6) | 0.034 |
| Current Depression | 184 | (13.9) | 519 | (23.4) | 457 | (32.8) | 158 | (39.9) | <0.001 |
| Current Anxiety | 261 | (19.7) | 614 | (27.7) | 488 | (34.9) | 148 | (37.5) | <0.001 |
| Current Arthritis = Yes (%) | 418 | (31.6) | 816 | (36.8) | 699 | (50.1) | 239 | (60.4) | <0.001 |
| Balance Poor = Yes (%) | 16 | (25.0) | 50 | (61.0) | 41 | (89.1) | (%) 9 | (100.0) | <0.001 |
| Current Back Pain = Yes (%) | 323 | (24.1) | 759 | (33.8) | 685 | (48.3) | 204 | (50.5) | <0.001 |
| Back Pain Limit Activities = Yes (%) | 175 | (54.2) | 516 | (68.0) | 551 | (80.4) | 179 | (87.7) | <0.001 |
| Work-related Activity = Yes (%) | 601 | 42.7 | 928 | 40.8 | 379 | 26.5 | 55 | 13.4 | <0.001 |
| Pain (%) |  |  |  |  |  |  |  |  | <0.001 |
| None | 640 | (41.5) | 603 | (23.7) | 214 | (13.2) | 48 | (10.5) |  |
| Slight | 697 | (45.2) | 1190 | (46.7) | 576 | (35.5) | 102 | (22.3) |  |
| Moderate | 186 | (12.1) | 674 | (26.4) | 677 | (41.7) | 199 | (43.5) |  |
| Severe | 17 | (1.1) | 78 | (3.1) | 142 | (8.7) | 86 | (18.8) |  |
| Extreme | 1 | (0.1) | 4 | (0.2) | 14 | (0.9) | 22 | (4.8) |  |
| Trouble Getting out of bed, a care, or a deep chair (%) |  |  |  |  |  |  |  |  | <0.001 |
| Normal | 795 | (56.7) | 574 | (25.5) | 122 | (8.6) | 10 | (2.5) |  |
| Slight | 541 | (38.6) | 1262 | (56.1) | 670 | (47.2) | 102 | (25.2) |  |
| Mild | 55 | (3.9) | 329 | (14.6) | 411 | (29.0) | 118 | (29.1) |  |
| Moderate | 10 | (0.7) | 80 | (3.6) | 194 | (13.7) | 114 | (28.1) |  |
| Severe | 0 | 0.0 | 3 | (0.1) | 21 | (1.5) | 61 | (15.1) |  |
| Problems with Balance and Walking |  |  |  |  |  |  |  |  | <0.001 |
| Normal | 988 | (70.5) | 472 | (21.0) | 52 | (3.7) | 2 | (0.5) |  |
| Slight | 394 | (28.1) | 1494 | (66.5) | 611 | (43.1) | 42 | (10.4) |  |
| Mild | 19 | (1.4) | 221 | (9.8) | 366 | (25.8) | 65 | (16.0) |  |
| Moderate | 0 | 0.0 | 56 | (2.5) | 373 | (26.3) | 215 | (53.1) |  |
| Severe | 0 | 0.0 | 5 | (0.2) | 16 | (1.1) | 81 | (20.0) |  |
| Suddenly stop or freeze when walking |  |  |  |  |  |  |  |  | <0.001 |
| Normal | 1316 | (93.9) | 1699 | (75.6) | 652 | (46.0) | 93 | (23.0) |  |
| Slight | 76 | (5.4) | 417 | (18.5) | 405 | (28.6) | 83 | (20.5) |  |
| Mild | 7 | (0.5) | 103 | (4.6) | 214 | (15.1) | 70 | (17.3) |  |
| Moderate | 2 | (0.1) | 27 | (1.2) | 127 | (9.0) | 89 | (22.0) |  |
| Severe | 0 | 0.0 | 2 | (0.1) | 20 | (1.4) | 70 | (17.3) |  |
| Walking Activities |  |  |  |  |  |  |  |  | <0.001 |
| Never | 54 | (3.8) | 130 | (5.7) | 150 | (10.5) | 145 | (35.3) |  |
| Seldom | 216 | (15.4) | 421 | (18.5) | 390 | (27.2) | 99 | (24.1) |  |
| Sometimes | 312 | (22.2) | 646 | (28.4) | 382 | (26.7) | 88 | (21.4) |  |
| Often | 825 | (58.6) | 1079 | (47.4) | 510 | (35.6) | 79 | (19.2) |  |
| Light sport and recreational activities |  |  |  |  |  |  |  |  | <0.001 |
| Never | 791 | (56.4) | 1318 | (58.1) | 993 | (69.7) | 337 | (82.0) |  |
| Seldom | 308 | (22.0) | 503 | (22.2) | 248 | (17.4) | 39 | (9.5) |  |
| Sometimes | 206 | (14.7) | 307 | (13.5) | 123 | (8.6) | 25 | (6.1) |  |
| Often | 98 | (7.0) | 141 | (6.2) | 61 | (4.3) | 10 | (2.4) |  |
| Moderate sport and recreational activities |  |  |  |  |  |  |  |  | <0.001 |
| Never | 931 | (66.2) | 1648 | (72.7) | 1200 | (84.1) | 382 | (93.2) |  |
| Seldom | 224 | (15.9) | 329 | (14.5) | 128 | (9.0) | 16 | (3.9) |  |
| Sometimes | 173 | (12.3) | 212 | (9.3) | 66 | (4.6) | 10 | (2.4) |  |
| Often | 78 | (5.5) | 79 | (3.5) | 33 | (2.3) | 2 | (0.5) |  |
| Strenuous sport and recreational activities |  |  |  |  |  |  |  |  | <0.001 |
| Never | 728 | (51.8) | 1415 | (62.4) | 1072 | (75.2) | 356 | (86.8) |  |
| Seldom | 218 | (15.5) | 353 | (15.6) | 142 | (10.0) | 18 | (4.4) |  |
| Sometimes | 261 | (18.6) | 316 | (13.9) | 142 | (10.0) | 18 | (4.4) |  |
| Often | 198 | (14.1) | 183 | (8.1) | 69 | (4.8) | 18 | (4.4) |  |
| Muscle strength |  |  |  |  |  |  |  |  | <0.001 |
| Never | 395 | (28.1) | 803 | (35.3) | 572 | (40.0) | 188 | (46.1) |  |
| Seldom | 381 | (27.1) | 604 | (26.6) | 377 | (26.4) | 102 | (25.0) |  |
| Sometimes | 445 | (31.7) | 610 | (26.8) | 338 | (23.7) | 85 | (20.8) |  |
| Often | 185 | (13.2) | 255 | (11.2) | 142 | (9.9) | 33 | (8.1) |  |

**Supplementary Table 4.** Post-hoc analyses of baseline sociodemographic and clinical factors at baseline across most likely cluster assignment for later disease duration >10 years.

|  | Most likely cluster assignment | | | | | | | | | |  |
| --- | --- | --- | --- | --- | --- | --- | --- | --- | --- | --- | --- |
|  | 1 |  | 2 |  | 3 |  | 4 |  | 5 |  | p |
| n | 330 |  | 750 |  | 671 |  | 261 |  | 58 |  |  |
| Mobility Impairment (%) |  |  |  |  |  |  |  |  |  |  | <0.001 |
| None | 234 | (70.9) | 67 | (8.9) | 6 | (0.9) | 0 | 0.0 | 0 | 0.0 |  |
| Slight | 93 | (28.2) | 574 | (76.5) | 101 | (15.1) | 3 | (1.1) | 0 | 0.0 |  |
| Moderate | 3 | (0.9) | 104 | (13.9) | 478 | (71.2) | 86 | (33.0) | 1 | (1.7) |  |
| Severe | 0 | 0.0 | 5 | (0.7) | 86 | (12.8) | 170 | (65.1) | 22 | (37.9) |  |
| Extreme | 0 | 0.0 | 0 | 0.0 | 0 | 0.0 | 2 | (0.8) | 35 | (60.3) |  |
|  |  |  |  |  |  |  |  |  |  |  |  |
| Sociodemographics |  |  |  |  |  |  |  |  |  |  |  |
| BMI (mean (SD)) | 24.54 | (3.9) | 26.03 | (4.9) | 26.67 | (5.4) | 27.02 | (5.6) | 27.04 | (6.8) | <0.001 |
| Education (mean (SD)) | 4.93 | (1.5) | 4.76 | (1.6) | 4.66 | (1.6) | 4.58 | (1.7) | 4.36 | (1.8) | 0.017 |
| Income (mean (SD)) | 4.55 | (1.5) | 4.22 | (1.6) | 3.96 | (1.6) | 3.64 | (1.7) | 4 | (1.7) | <0.001 |
| Age (mean (SD)) | 67.09 | (7.8) | 65.96 | (7.7) | 67.64 | (8.3) | 69.31 | (9.1) | 72.22 | (8.2) | <0.001 |
| Sex = Female (%) | 156 | (48.1) | 338 | (46.2) | 295 | (45.2) | 102 | (40.3) | 24 | (42.9) | 0.411 |
| Race = Non-White (%) | 11 | (3.4) | 17 | (2.3) | 11 | (1.7) | 11 | (4.3) | 2 | (3.6) | 0.164 |
| Employment (%) |  |  |  |  |  |  |  |  |  |  |  |
| Full | 33 | (10.2) | 63 | (8.7) | 34 | (5.2) | 2 | (0.8) | 0 | 0.0 | <0.001 |
| Part-time | 23 | (7.1) | 46 | (6.3) | 29 | (4.5) | 3 | (1.2) | 2 | (3.6) | 0.008 |
| Retired | 256 | (79.3) | 582 | (80.3) | 552 | (85.1) | 227 | (90.1) | 52 | (92.9) | <0.001 |
| Unemployed | 11 | (3.4) | 34 | (4.7) | 34 | (5.2) | 20 | (7.9) | 2 | (3.6) | 0.152 |
|  |  |  |  |  |  |  |  |  |  |  |  |
| Clinical factors |  |  |  |  |  |  |  |  |  |  |  |
| Veteran (%) | 47 | (14.5) | 112 | (15.4) | 102 | (15.7) | 41 | (16.3) | 10 | (17.9) | 0.961 |
| OFF Episodes = Yes (%) | 5 | (41.7) | 23 | (71.9) | 17 | (68.0) | 4 | (57.1) | 0 | 0.0 | 0.238 |
| Current Medication for PD = Yes (%) | 303 | (97.4) | 701 | (97.9) | 632 | (98.0) | 237 | (96.3) | 55 | (98.2) | 0.642 |
| Current Depression | 54 | (18.4) | 167 | (25.5) | 193 | (32.1) | 86 | (39.3) | 27 | (52.9) | <0.001 |
| Current Anxiety | 63 | (21.6) | 182 | (27.8) | 219 | (36.4) | 95 | (43.0) | 19 | (37.3) | <0.001 |
| Current Arthritis = Yes (%) | 86 | (29.5) | 268 | (40.9) | 296 | (49.1) | 117 | (52.9) | 28 | (54.9) | <0.001 |
| Balance Poor = Yes (%) | 7 | (41.2) | 10 | (71.4) | 13 | (81.2) | 5 | (100.0) | 1 | (100.0) | 0.044 |
| Current Back Pain = Yes (%) | 82 | (27.5) | 263 | (39.4) | 290 | (47.8) | 106 | (46.1) | 20 | (37.7) | <0.001 |
| Back Pain Limit Activities = Yes (%) | 48 | (58.5) | 194 | (73.8) | 234 | (80.7) | 86 | (81.1) | 13 | (65.0) | <0.001 |
| Work-related Activity = Yes (%) | 109 | 37.1 | 211 | 31.7 | 120 | 20.7 | 30 | 13.2 | 7 | 13.2 | <0.001 |
| Pain (%) |  |  |  |  |  |  |  |  |  |  | <0.001 |
| None | 122 | (37.0) | 160 | (21.4) | 93 | (13.9) | 24 | (9.2) | 5 | (8.8) |  |
| Slight | 158 | (47.9) | 349 | (46.7) | 223 | (33.4) | 59 | (22.6) | 15 | (26.3) |  |
| Moderate | 49 | (14.8) | 212 | (28.3) | 272 | (40.7) | 120 | (46.0) | 26 | (45.6) |  |
| Severe | 1 | (0.3) | 22 | (2.9) | 73 | (10.9) | 46 | (17.6) | 11 | (19.3) |  |
| Extreme | 0 | 0.0 | 5 | (0.7) | 7 | (1.0) | 12 | (4.6) | 0 | 0.0 |  |
| Trouble Getting out of bed, a care, or a deep chair (%) |  |  |  |  |  |  |  |  |  |  | <0.001 |
| Normal | 110 | (36.5) | 93 | (14.1) | 34 | (5.9) | 7 | (3.1) | 0 | 0.0 |  |
| Slight | 140 | (46.5) | 342 | (51.9) | 224 | (38.8) | 48 | (21.3) | 4 | (7.5) |  |
| Mild | 41 | (13.6) | 154 | (23.4) | 187 | (32.4) | 70 | (31.1) | 9 | (17.0) |  |
| Moderate | 9 | (3.0) | 68 | (10.3) | 123 | (21.3) | 72 | (32.0) | 12 | (22.6) |  |
| Severe | 1 | (0.3) | 2 | (0.3) | 9 | (1.6) | 28 | (12.4) | 28 | (52.8) |  |
| Problems with Balance and Walking |  |  |  |  |  |  |  |  |  |  | <0.001 |
| Normal | 159 | (52.8) | 69 | (10.5) | 4 | (0.7) | 1 | (0.4) | 0 | 0.0 |  |
| Slight | 122 | (40.5) | 371 | (56.3) | 167 | (28.9) | 13 | (5.8) | 0 | 0.0 |  |
| Mild | 15 | (5.0) | 161 | (24.4) | 171 | (29.6) | 23 | (10.2) | 1 | (1.9) |  |
| Moderate | 5 | (1.7) | 51 | (7.7) | 218 | (37.8) | 147 | (65.3) | 10 | (18.9) |  |
| Severe | 0 | 0.0 | 7 | (1.1) | 17 | (2.9) | 41 | (18.2) | 42 | (79.2) |  |
| Suddenly stop or freeze when walking |  |  |  |  |  |  |  |  |  |  | <0.001 |
| Normal | 228 | (75.7) | 316 | (48.0) | 169 | (29.3) | 38 | (16.9) | 10 | (18.9) |  |
| Slight | 59 | (19.6) | 205 | (31.1) | 142 | (24.6) | 31 | (13.8) | 8 | (15.1) |  |
| Mild | 12 | (4.0) | 93 | (14.1) | 110 | (19.1) | 32 | (14.2) | 5 | (9.4) |  |
| Moderate | 2 | (0.7) | 44 | (6.7) | 138 | (23.9) | 83 | (36.9) | 3 | (5.7) |  |
| Severe | 0 | 0.0 | 1 | (0.2) | 18 | (3.1) | 41 | (18.2) | 27 | (50.9) |  |
| Walking Activities |  |  |  |  |  |  |  |  |  |  | <0.001 |
| Never | 6 | (2.0) | 37 | (5.5) | 60 | (10.3) | 63 | (27.5) | 35 | (66.0) |  |
| Seldom | 55 | (18.6) | 142 | (21.2) | 176 | (30.3) | 68 | (29.7) | 6 | (11.3) |  |
| Sometimes | 80 | (27.0) | 186 | (27.8) | 154 | (26.5) | 59 | (25.8) | 7 | (13.2) |  |
| Often | 155 | (52.4) | 304 | (45.4) | 191 | (32.9) | 39 | (17.0) | 5 | (9.4) |  |
| Light sport and recreational activities |  |  |  |  |  |  |  |  |  |  | <0.001 |
| Never | 158 | (53.2) | 383 | (57.4) | 401 | (69.4) | 182 | (79.5) | 46 | (86.8) |  |
| Seldom | 70 | (23.6) | 145 | (21.7) | 106 | (18.3) | 26 | (11.4) | 4 | (7.5) |  |
| Sometimes | 45 | (15.2) | 96 | (14.4) | 49 | (8.5) | 14 | (6.1) | 2 | (3.8) |  |
| Often | 24 | (8.1) | 43 | (6.4) | 22 | (3.8) | 7 | (3.1) | 1 | (1.9) |  |
| Moderate sport and recreational activities |  |  |  |  |  |  |  |  |  |  | <0.001 |
| Never | 198 | (67.1) | 484 | (72.5) | 481 | (83.1) | 207 | (90.4) | 51 | (96.2) |  |
| Seldom | 42 | (14.2) | 103 | (15.4) | 56 | (9.7) | 13 | (5.7) | 1 | (1.9) |  |
| Sometimes | 37 | (12.5) | 61 | (9.1) | 35 | (6.0) | 7 | (3.1) | 1 | (1.9) |  |
| Often | 18 | (6.1) | 20 | (3.0) | 7 | (1.2) | 2 | (0.9) | 0 | 0.0 |  |
| Strenuous sport and recreational activities |  |  |  |  |  |  |  |  |  |  | <0.001 |
| Never | 167 | (56.8) | 429 | (64.1) | 438 | (75.8) | 195 | (85.5) | 48 | (90.6) |  |
| Seldom | 43 | (14.6) | 94 | (14.1) | 60 | (10.4) | 16 | (7.0) | 3 | (5.7) |  |
| Sometimes | 58 | (19.7) | 102 | (15.2) | 57 | (9.9) | 10 | (4.4) | 1 | (1.9) |  |
| Often | 26 | (8.8) | 44 | (6.6) | 23 | (4.0) | 7 | (3.1) | 1 | (1.9) |  |
| Muscle strength |  |  |  |  |  |  |  |  |  |  | 0.343 |
| Never | 107 | (36.1) | 252 | (37.6) | 243 | (42.0) | 101 | (44.1) | 25 | (47.2) |  |
| Seldom | 74 | (25.0) | 161 | (24.0) | 152 | (26.3) | 56 | (24.5) | 12 | (22.6) |  |
| Sometimes | 82 | (27.7) | 180 | (26.9) | 131 | (22.6) | 47 | (20.5) | 9 | (17.0) |  |
| Often | 33 | (11.1) | 77 | (11.5) | 53 | (9.2) | 25 | (10.9) | 7 | (13.2) |  |
